# Alterations in gene expression in the spinal cord of mice lacking *Nfix*

**DOI:** 10.1186/s13104-020-05278-w

**Published:** 2020-09-16

**Authors:** Elise Matuzelski, Alexandra Essebier, Lachlan Harris, Richard M. Gronostajski, Tracey J. Harvey, Michael Piper

**Affiliations:** 1 School of Biomedical Sciences, The Faculty of Medicine, The University of Queensland, Brisbane, QLD 4072 Australia; 2 School of Chemistry and Molecular Bioscience Sciences, The Faculty of Science, The University of Queensland, Brisbane, QLD 4072 Australia; 3 Department of Biochemistry, Program in Genetics, Genomics and Bioinformatics, Center of Excellence in Bioinformatics and Life Sciences, State University of New York at Buffalo, Buffalo, NY USA; 4 Queensland Brain Institute, The University of Queensland, Brisbane, QLD 4072 Australia

**Keywords:** Nuclear Factor One, NFIX, Spinal cord, Microarray

## Abstract

**Objective:**

Nuclear Factor One X (NFIX) is a transcription factor expressed by neural stem cells within the developing mouse brain and spinal cord. In order to characterise the pathways by which NFIX may regulate neural stem cell biology within the developing mouse spinal cord, we performed an microarray-based transcriptomic analysis of the spinal cord of embryonic day (E)14.5 *Nfix*^*−/−*^ mice in comparison to wild-type controls.

**Data description:**

Using microarray and differential gene expression analyses, we were able to identify differentially expressed genes in the spinal cords of E14.5 *Nfix*^*−/−*^ mice compared to wild-type controls. We performed microarray-based sequencing on spinal cords from n = 3 E14.5 *Nfix*^*−/−*^ mice and n = 3 E14.5 *Nfix*^+*/*+^ mice. Differential gene expression analysis, using a false discovery rate (FDR) *p*-value of *p* < 0.05, and a fold change cut-off for differential expression of >  ± 1.5, revealed 1351 differentially regulated genes in the spinal cord of *Nfix*^*−/−*^ mice. Of these, 828 were upregulated, and 523 were downregulated. This resource provides a tool to interrogate the role of this transcription factor in spinal cord development.

## Objective

Transcription factors play a central role in promoting stem cell biology within the developing embryo. For example, the Nuclear Factor One (NFI) transcription factors regulate fetal muscle gene expression [1], retinal development [2], and dorsal telencephalic formation [3, 4]. NFIX specifically promotes neural stem cell differentiation within the developing neocortex, hippocampus and cerebellum [5–8], as well as within the adult forebrain neurogenic niches [9, 10]. Mechanistically, NFIs have been shown to drive differentiation within the nervous system via the activation of lineage-specific gene expression patterns, as well as via the repression of stem cell self-renewal genes [11, 12].

NFIs also contribute to mouse spinal cord development. Mice lacking either *Nfia* or *Nfib* exhibit reduced expression of the astrocyte marker GFAP at E18.5 [13]. Moreover, NFIA has been proposed to mediate the switch in neural progenitor cell activity, promoting progression down a glial lineage, rather than the neural lineage [13]. NFIA has more recently been shown to interact with SOX9 in order to promote the expression of astrocytic genes, including *Apcdd1* and *Mmd2* [14]. We have also recently shown that *Nfix*-deficient mice also exhibit delayed astrocytic differentiation within the mouse spinal cord, with *Nfix* expression reliant, at least in part, on the activity of NFIB [15]. However, although we analysed the expression of NFIX within the developing mouse spinal cord, as well as the broad cellular phenotypes of the spinal cord in *Nfix*^*−/−*^ mice, we did not perform a global analysis of those genes that were misregulated in *Nfix* mutant mice. To gain a more comprehensive understanding of the transcriptional landscape controlled by NFIX within the spinal cord, here we performed microarray analysis on E14.5 wild-type and *Nfix*^*−/−*^ spinal cords, the age at which the expression of NFIX by neural progenitor cells lining the spinal central canal is highest [15].

## Data description

The rationale behind this profiling experiment was to understand transcriptional changes arising in the developing spinal cord in the absence of *Nfix.* To do this, *Nfix*^*−/−*^ mice [16] and their wild-type littermates were used. These mice were maintained on a C57Bl/6J genetic background. Heterozygous male and female mice were placed together overnight to produce time-mated litters. The presence of a vaginal plug the following day indicated a successful mating was likely, and this was designated E0.5. Polymerase chain reactions (PCR) confirmed the genotypes of each mouse. PCR primers are available on request. Pregnant dams were euthanized by cervical dislocation. Embryos were removed and placed on ice. Whole spinal cords were dissected from E14.5 *Nfix*^*−/−*^ and wild-type mouse embryos and snap frozen using dry ice. An RNeasy Micro Kit (QIAGEN) was used to extract total RNA from these samples, which were then diluted to 80 ng/μL and sent to the Ramaciotti Centre (University of New South Wales, Australia) for microarray analysis on a Mouse Transcriptome Array 2.0ST (Affymetrix). The Ramaciotti Centre performed sample quality control at three points in the process; (1) first-cycle cRNA after clean up (spectrophotomoter and Bioanalyzer), (2) second-cycle single-stranded cDNA after clean up (spectrophotomoter and Bioanalyzer), and (3) fragmented and labelled cDNA (Bioanalyzer and gel-shift assay). All the samples passed these checkpoints. For the Bioanalyzer analyses, all samples reported an RNA integrity number > 8. For the spectrophotometer analyses, the OD 260/280 ratio was used to measure the purity of the RNA; all samples had values ~ 2. Finally, the QC metric pos_vs_neg_auc value was used to determine the quality of the array data. Values above 0.8 identify the data as within the bounds for the threshold test; all our samples were > 0.91.

Raw microarray data in the form of.CEL files were received from the Ramaciotti Centre and initially processed by running an SST-RMA (gene level) analysis on all samples using the Affymetrix Expression Console software (build 1.4.1.46). The resulting.CHP files were then imported into the Affymetrix Transcription Analysis Console (TAC) software and sample files were compared in differential gene expression analyses between *Nfix*^*−/−*^ and wild-type samples. Algorithm parameters selected included: genome version mm10, one-way between-subject ANOVA (unpaired) and ANOVA *p*-value (condition pair) < 0.05. Differential gene expression analyses were performed using Affymetrix Transcription Analysis Console (TAC) software. Gene expression levels between *Nfix*^*−/−*^ and wild-type samples were compared using a one-way between-subject ANOVA (unpaired) and corrected for FDR. A *p-*value < 0.05 denoted a statistically significant difference in gene expression between samples. A fold change cut-off for differential expression of >  ± 1.5 was also used to stratify differentially expressed genes.

Table 1 presents data repositories arising from this work. The raw sequencing data are available from GEO (Data set 1) [17]. Differential gene analysis revealed that 828 genes were upregulated in the E14.5 spinal cord of *Nfix*^*−/−*^ mice in comparison to controls, whereas 523 genes were downregulated in the spinal cord of *Nfix*^*−/−*^ mice in comparison to controls (Data set 2) [18].

**Table 1:** Overview of data files

Label	Name of data file	File types (file extension)	Data repository and identifier (DOI or accession number)
Data file 1	Expression data from embryonic mouse spinal cords from NFIX knockout and wild-type mice	CEL file	https://identifiers.org/geo:GSE155327 [17]
Data file 2	Differentially regulated genes E14.5 spial cord NFIX KO vs. WT	Excel spreadsheet (.xlsx)	https://doi.org/10.6084/m9.figshare.12735860.v1 [18]

## Limitations

This work complements our recently published work [15], and provides additional, global insights into the changes in transcriptional programs evident in the spinal cord in the absence of *Nfix.* While valuable, there are a number of limitations that need to be considered when interpreting these data. Firstly, our analysis of transcriptional changes in the spinal cord of *Nfix*^*−/−*^ mice was only conducted at one age. Investigating further embryonic and postnatal ages would provide more context around the role of NFIX in regulating genesis of the spinal cord. Secondly, although the expression of NFIX is prominent within the neural stem cells of the spinal cord at this age [15], NFIX is also expressed by other cell types within the spinal cord. As such, interpretation of the data needs to occur with this caveat in mind. The use of single cell RNA-sequencing in future could be one way to circumvent this limitation, and to garner a more nuanced view of how NFIX regulates the activity of different cell types within the spinal cord. We also only analysed potential targets at an mRNA level. Assessing changes at the protein level, using either western blotting or immunohistochemistry, would be a powerful complementary approach. Finally, our data alone do not enable us to discern direct versus indirect targets of NFIX. This could be addressed in future using approaches such as chromatin immunoprecipitation and sequencing, which we have recently used to parse the roles of different NFI proteins within the postnatal cerebellum [5].

## Data Availability

The raw sequencing data described in this Data note can be freely and openly accessed through GEO (Data file 1) [17]. Data file 2 is available on Figshare (https://figshare.com) [18]. Please see Table 1 for links to the data.

## Abbreviations

NFI: Nuclear Factor One
NFIA: Nuclear Factor One A
NFIB: Nuclear Factor One B
NFIX: Nuclear Factor One X
